# Selenium species transforming along soil–plant continuum and their beneficial roles for horticultural crops

**DOI:** 10.1093/hr/uhac270

**Published:** 2022-12-02

**Authors:** Qingxue Guo, Jianhui Ye, Jianming Zeng, Liang Chen, Helena Korpelainen, Chunyang Li

**Affiliations:** College of Life and Environmental Sciences, Hangzhou Normal University, Hangzhou 311121, China; College of Agriculture and Biotechnology, Zhejiang University, Hangzhou 310058, China; Tea Research Institute, Chinese Academy of Agricultural Sciences, Hangzhou 310008, China; Tea Research Institute, Chinese Academy of Agricultural Sciences, Hangzhou 310008, China; Department of Agricultural Sciences, Viikki Plant Science Centre, University of Helsinki, P.O. Box 27, FI-00014, Finland; College of Agriculture and Biotechnology, Zhejiang University, Hangzhou 310058, China

## Abstract

Selenium (Se) acquirement from daily diet can help reduce the risk of many diseases. The edible parts of crop plants are the main source of dietary Se, while the Se content in crops is determined by Se bioavailability in soil. We summarize recent research on the biogeochemical cycle of Se driven by specific microorganisms and emphasize the oxidizing process in the Se cycle. Moreover, we discuss how plant root exudates and rhizosphere microorganisms affect soil Se availability. Finally, we cover beneficial microorganisms, including endophytes, that promote crop quality and improve crop tolerance to environmental stresses. Se availability to plants depends on the balance between adsorption and desorption, reduction, methylation and oxidation, which are determined by interactions among soil properties, microbial communities and plants. Reduction and methylation processes governed by bacteria or fungi lead to declined Se availability, while Se oxidation regulated by Se-oxidizing microorganisms increases Se availability to plants. Despite a much lower rate of Se oxidization compared to reduction and methylation, the potential roles of microbial communities in increasing Se bioavailability are probably largely underestimated. Enhancing Se oxidation and Se desorption are crucial for the promotion of Se bioavailability and uptake, particularly in Se-deficient soils. Beneficial roles of Se are reported in terms of improved crop growth and quality, and enhanced protection against fungal diseases and abiotic stress through improved photosynthetic traits, increased sugar and amino acid contents, and promoted defense systems. Understanding Se transformation along the plant–soil continuum is crucial for agricultural production and even for human health.

## Introduction

Selenium (Se) is a naturally occurring trace element with a significant importance for human health. Crucial functions of different selenoproteins detected in human bodies have clearly demonstrated the fundamental role of Se for life [1]. The selenoproteins involved in a series of enzyme systems, such as glutathione peroxidase and thioredoxin reductase, show functions as anti-cancer agents, in the inhibition of HIV development and in other related immune system disorders [2]. The distribution of Se is uneven on the surface of the Earth, leading to both Se-deficient and seleniferous regions [3, 4]. Their concentration in most soils ranges from 0.01 to 2.0 mg kg^−1^with an average Se content of 0.4 mg kg^−1^ [5]. An estimated 15% of the global population is Se-deficient, i.e. having a lower daily Se intake than 26–34 μg per day [6]. However, a high bioaccumulation of selenium (>400 μg per day) can also be toxic to humans [5, 7]. Therefore, both Se deficiency and toxicity are emerging issues that attract the attention of researchers worldwide.

**Figure 1:**
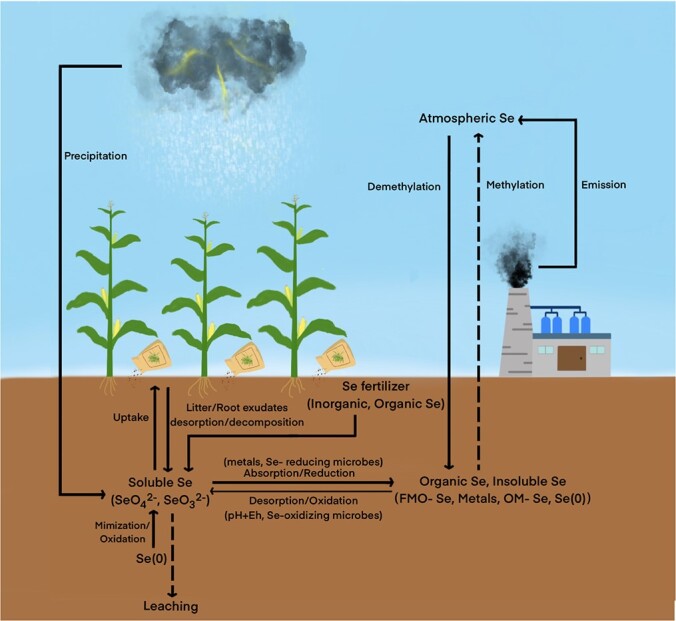
A framework of the selenium (Se) cycle. The Se availaibility depends on Se oxyanion (SeO_4_^2−^ and SeO_3_^2−^) contents in soil, but is deterimined by adsorption and desorption, reduction, methylation, and oxidation. Soil organic matter, pH, and other properties mainly affect adsorption and desorption, while reduction, methylation, and oxidation processes are mainly driven by microbical communities. Crops can affect Se avabilibility through root exudates and species-specific microbical communities. FMO-Se: Se absorbed by soil metals like Fe and Mn. OM-Se: Se absorbed by soil organic matter.

For instance, volcanic eruptions and anthropogenic sources generate atmospheric Se (Fig. 1) [8]. Se in the atmosphere, and in marine and terrestrial ecosystems can be transported and transformed into different forms via diverse pathways [8]. Different Se forms are then affected by soil properties, transformed by microorganisms and taken up by plants (Fig. 1). Several studies have considered the crucial roles of microorganisms in influencing the biogeochemical cycle of Se (reduction, methylation, or oxidation), which alter Se speciation and contents [9–11]. However, the results of most studies have utilized culture-based approaches, and individual microorganisms involved in Se species transformation in Se cycling processes have been identified. Information concerning interactions/connections among or within functional guilds (reduction, methylation, and oxidation) is still lacking. Nowadays, the crucial role of plants in driving soil nutrient cycles has received much attention in forest, grassland, and agricultural ecosystems [12, 13]. Because Se is critical to crop plants, understanding the migration and transformation of Se in the plant–soil continuum and plant uptake of Se, as affected by microorganisms, is of particular importance (Fig. 1).

In this review, the effects of soil properties, microorganisms, and plants on Se species and cycle are discussed. It is important to know the underlying mechanisms that affect the contents of selenate, selenite, and some organic Se species, particularly in Se-deficiency areas, as they are the available forms for absorption by crop plants. In addition, more studies are focusing on the beneficial roles of Se in terms of improving plant growth and crop quality, and enhancing protection against fungal diseases or abiotic stresses. Therefore, how Se affects plant growth, quality, and defense is also discussed.

## Selenium species and fractions in soil

Selenate (SeO_4_^2−^), selenite (SeO_3_^2−^), elemental Se (Se^0^), and selenide (Se^2−^) are four oxidation states of Se in soil. Selenate and selenite can be acquired by plants due to their high solubility in soil. The inorganic Se (SeO_4_^2−^, SeO_3_^2−^ and Se^0^) is the main form distributed in soil, but organic forms, such as selenomethionine (SeMet), selenocysteine (SeCys), and methylselenocysteine are also present [14]. Se fractions are separated into soluble Se, exchangeable Se, Fe-Mn oxide-bound Se, organic matter-bound Se, and residual Se [15]. Changes among different valent states affect Se fractions due to their differences in migration mobility and binding intensity, further affecting Se bioavailability in soil. Soluble Se fractions in soil include soluble organic Se and Se oxyanions (SeO_4_^2−^ and SeO_3_^2−^) [16]. Some Se species can be bound by metal ion oxides like iron, manganese, and organic matter in soil to form Fe-Mn oxide-bound Se and organic-bound Se [17, 18]. The bound Se fractions are suggested as potential Se pools at a given moment, because these fractions can be released [16]. Most Se in soil is not available for plants [15, 19, 20]. The distribution pattern of Se fractions is at a dynamic equilibrium state [21], which is regulated by sorption/desorption, precipitation/dissolution, and oxidation/reduction processes. The strength of these transformation processes is controlled by soil properties, such as soil moisture, pH, redox conditions, organic matter, and microbial functions.

Inorganic Se(VI) and Se(IV) are the main Se fractions that determine the bioavailable Se content in soil. Selenate is more soluble and available to plants, whereas selenite is efficiently absorbed by different soil constituents, especially clay and metal-oxides, thus being less soluble in soil. The transformation between Se(VI) and Se(IV) largely depends on soil pH and redox potential (Eh). Se(VI) is the dominant Se form in well-aerated alkaline soils but it is readily converted to selenite along decreasing pH in neutral and acid soils [22]. The increased pH facilitates desorption of Se from metals and increases the availability of soil Se [23]. However, the contents of insoluble Se(0) and Se(-II) (Se^2−^) increase under reduced conditions (for example Eh < −200 mV), resulting in low Se bioavailability [22, 24]. In general, acid soils release less selenium than alkaline ones, because selenite is easily fixed by metals like iron hydroxides. In alkaline soils, selenite is oxidized and it forms selenate [16, 25].

Different sorptive behaviors of SeO_4_^2−^ and SeO_3_^2−^ adsorbed onto geocolloidal phases of soil are due to their different mechanisms, including inner- and outer-sphere complexation [26, 27]. Outer-sphere adsorption formed by weak electrostatic forces (via hydration shell) between SeO_4_^2−^ and the functional guilds of soil sorbent phases (like metals) is usually reversible [16, 26]. Inner-sphere complexes formed by covalent bonding between the ions and functional groups (directly via the Se atom) result in stronger sorption [16, 27]. As mentioned above, soil metals, such as iron and aluminum, are other crucial factors that affect Se fractions, because of their extensive chelating ability in different soil pH and Eh conditions [16, 28].

Soil clay and organic matter contents are also key factors in regulating Se transformation in soils [25, 29]. Selenium oxyanions can be adsorbed by positively charged clay minerals [17]. Twidwell (2011) [30] reported a stronger Se(IV) adsorption by clay compared to Se(VI) because of differences in inner- and outer-sphere. Previous studies have reported that even over half of the total soil Se is bound with and/or incorporated into organic matters [18, 31]. Other soil conditions like moisture and oxygen are also important factors in determining Se fractions by affecting metals, Eh, pH, dissolved organic matter, and microbial activity [31]. Decreasing soil Eh but increasing dissolved organic carbon under water-saturated conditions result in the reduction of Se(VI) into low valences like Se(IV), Se(0), or Se(-II) [32]. It is obvious that the Se forms in soil are largely dependent on soil pH, organic matter, Eh, and texture. These factors in combination or alone could affect reactions like sorption/desorption, precipitation/dissolution, and oxidation/reduction processes, and determine the fate and behavior of Se in soil.

## Selenium biochemical cycle driven by microorganisms

The composition and structure of soil microbial communities affect the availability of multiple nutrients by determining their cycling processes. Since the first discovery of selenite demand for the growth of *Escherichia coli*, there has been a growing interest in the biochemical role of Se in microbes [33]. Wang *et al.* (2022) [19] reviewed that in the presence of metals, such as Pb, Zn, Cd, Cu, Ag, and Bi, soil microorganisms can reduce Se (IV) to corresponding nanostructured metal selenides. As mentioned above, some specific bacterial and fungal species in soil are obligated to transform Se among different valent states and, thus, affect its bioavailability by reduction/oxidation and methylation/demethylation during the biogeochemical cycle of Se.

### Selenium reduction and methylation processes

The reduction and methylation processes by environmental microorganisms are well studied due to recent interest to remove Se toxicity in selenium contaminated environments or high selenium soils [34]. Many bacteria have been successfully isolated and proved to transform Se oxyanions to insoluble and less bioavailable Se(0) and Se(-II) through assimilatory and/or dissimilatory reduction pathways [9, 35]. Different studies have demonstrated that the reduction processes occur either in the periplasmic space (intracellularly) or extracellularly [36, 37]. The functioning of a series of reductases, such as nitrate reductases, respiratory selenate reductases and fumarate reductases, are well characterized in different Se-reducing bacteria [19, 38].

Selenate and selenite are first transported into bacterial cells and then they go through assimilatory reduction to selenocysteine or selenomethionine, which can be incorporated into proteins during bacterial growth. The dissimilatory Se-reducing bacteria can obtain metabolic energy by reducing SeO_3_^2−^ and SeO_4_^2−^ to selenium nanoparticles (Se^0^), and they are considered to remove Se oxyanions more efficiently than assimilatory Se-reducing bacterial species [39, 40]. Several excellent reviews have discussed the mechanisms of Se reduction [19, 38–40]. For example, Eswayah *et al.* (2016) [39] and Wang *et al.* (2022) [19] reviewed the reduction of different Se species through diverse microbes, including bacteria and archaea, in different environments. Several new bacterial isolates, such as *Chitinophaga* sp. and *Comamonas testosteroni*, from paddy soil can transform selenite to elemental Se nanoparticles with different reducing rates under aerobic conditions [35]. Bacteria *Bacillus selenitireducens* and *Thiobacillus ferrooxidans* can generate selenide by reducing Se(0) [41, 42]. Some bacteria, such as *T. ferrooxidans* and *Desulfovibrio desulfuricans* subsp. *aestuarii*, are capable of reducing both sulphur and selenium [41, 43].

The effects of fungal species on Se behavior have recently been explored [44]. Six aerobic fungi *Pyrenochaeta* sp., *Plectosphaerella cucumerina, Paraconiothyrium sporulosum*, *Acremonium strictum, Stagonospora sp.,* and *Alternaria alternata* have showed their ability to reduce Se oxyanions to selenium nanoparticles or volatile selenium compounds [9]. However, the Se reduction processes of *P. sporulosum* and *Stagonospora* sp. were simultaneously accompanied by the biomineralization process of mycogenic Mn(II) oxidation to Mn oxides [45]. Liang *et al.* (2019) [46] also reported that four fungal species (*Aureobasidium pullulans*, *Mortierella humilis*, *Trichoderma harzianum, and Phoma glomerata*) were able to reduce SeO_3_^2−^and SeO_4_^2−^ to Se nanoparticles, and *P. glomerata* could precipitate elemental Se intracellularly and extracellularly when grown with selenite [47]. However, molecular mechanisms of these Se-reducing fungi need further research.

Different studies have confirmed that microorganisms govern the Se methylation process by transforming SeO_3_^2−^ and SeO_4_^2−^ to volatile compounds, such as dimethyl selenide and dimethyl diselenide [10, 39]. Thus, these microbes converting Se forms play an important role in Se cycling and may provide an efficient detoxification mechanism for selenium contaminated soil. The predominant Se-methylating bacteria and fungi are well summarized by Eswayah *et al.* (2016) [39]. The six fungal species mentioned in Rosenfeld *et al.* (2017) [10] are able to remove at least 15–20% of the supplied Se via volatilization.

### Selenium-oxidizing bacteria

Compared to the well-studied reduction and methylation processes, microbial Se oxidation has received less attention. To our knowledge, there are no studies conducted to assess the oxidation of Se(-II) and Se(0) in anoxic environments. Wells and Stolz (2020) [40] have suggested that having the expensive Na_2_Se or highly toxic H_2_Se as a source of Se(-II) was one principal obstacle. Some early studies have demonstrated that certain microbes are capable of aerobic oxidation of Se(0) and SeO_3_^2−^ [48, 49], and the oxidation is determined by the type of microbes [50]. However, the oxidation of Se(0) to SeO_3_^2−^ and SeO_4_^2−^occurs at relatively low rates [40, 50]. In contrast, the Se reducing rates are much more rapid [40]. For example, approximately 51% of China has Se-deficient soil [51]. Organic or inorganic forms of Se fertilizers are now widely applied in agriculture to enhance plants’ Se content [32, 52], whereas a maximum of 10% of inorganic Se fertilizers can be acquired by plants [52]. The bioavailability of Se dramatically declines through adsorption with organic matters and metals, and through reduction and methylation processes as reviewed above. Therefore, the potential roles of soil microorganisms in increasing the bioavailability of Se but reducing the environmental risk of inorganic Se fertilizers are important.

For both the control and optimization of Se oxidation, there is an increasing interest in understanding the underlying microbiological processes, including the biochemical fundamentals of the oxidation reactions. The Se-oxidizing bacteria have been reported to increase soil SeO_3_^2−^ and SeO_4_^2−^ contents although at low rates [49, 53, 54]. For instance, the heterotrophic bacterium *Bacillus megaterium* was discovered to oxidize elemental Se to selenite [49]. Then, heterotrophic and autotrophic oxidation of Se(0) yielding both SeO_3_^2−^ and SeO_4_^2−^ were found, which suggested that diverse soil microbes probably have a Se oxidation ability. A recent study clearly demonstrated that four strains of bacteria (*Dyella* spp. LX-1 and LX-66, and *Rhodanobacter* spp. LX-99 and LX-100) isolated from seleniferous soil can oxidize Se(0) to Se(IV) and dramatically increase water-soluble Se and exchangeable Se fractions in soil [53]. More importantly, the authors firstly found the oxidation of organic Se (selenomethionine and selenocystine) to Se(IV) with a higher oxidation efficiency at pH 8.56 than at pH 5.25 [53].

We have discussed that high soil pH oxidizes selenite to form selenate and also facilitates the desorption of Se from metals and increases Se availability in soil. High pH may enhance functions of these Se-oxidizing bacteria [54]. Several Se-oxidizing bacteria can also be found: the major oxidation product of Se(0) by *Thiobacillus* ASN-1 and *Leptothrix* MnB1 is Se(VI) rather than Se(IV) [55] (Dowdle and Oremland 1998), while the bacterial strain *Agrobacterium* sp. T3F4 mainly oxidizes Se(0) to Se(IV) [54]. These different Se oxidization products by diverse bacteria isolated from soil suggest the presence of complex processes of selenium species, for which the enzymes and pathways have not yet been reported. In addition, selenium is always bound with other metals, and chemical changes caused by bacteria may also induce Se transformations. For example, in the metabolic oxidation process of copper selenide by *T. ferrooxidans*, Se(-II) is transformed to Se(0) [48].

To date, all studies investigating Se-oxidation have used culture-based methods with aerobic bacterial species. Biological cycling of different nutrients are driven and determined by cooperation of different functional microbial groups. Although the oxidation rate of a bacteria-oxidizing strain is much lower than the reduction rate, the cooperation among diverse oxidizing species and other microorganisms probably has a great effect on the Se oxidization.

### Selenium availability promoted by fungi

Arbuscular mycorrhizal fungi (AMF) form symbiotic relationships with the majority of plant species and enhance the nutrient acquisition and transport of hosts. The added selenite and selenate fertilizers are easily absorbed or reduced as mentioned above. However, Li *et al.* (2020) [56] firstly found that the incubation of two AMF species, *Funneliformis mosseae* and *Glomus versiforme*, increased the concentration of available Se forms and facilitated Se uptake by crops. Another important result of Li *et al.* (2020) [56] is that the two AMF species also significantly increased Se bioavailability in soil without Se addition. While Se reduction is primarily physically associated with the fungal hyphae [9], information regarding the increasing selenite or selenate transformation to Se(0) is lacking. No studies have reported oxidative reactions of Se by fungi. However, AM fungi release diverse hyphae compounds to recruit a diversity of microbes in the hyphosphere, thus affecting nutrient cycling [57,58]. It is supposed that Se-oxidizing bacterial species may be recruited by AM fungi, which would increase the soil Se bioavailability. The inoculation of *F. mosseae* and *G. versiforme* were found to increase the relative abundance of Firmicutes, which led to a high content of available Se [59]. The successfully isolated Se-oxidizing *Bacillus* species belong to Firmicutes. However, the effects of environmental conditions, such as temperature, pH and water, on the activities of Se-oxidizing microorganisms have not been explored.

Besides AM fungi, diverse fungal species are expected to increase Se bioavailability. An application of Se-enriched organic plant material has been recommended as substitutes for inorganic Se to solve environmental problems [52]. Selenate and selenite species in soil are the most common forms used by crops and they are easily transformed to organic forms, such as selenomethionine, selenocystine, and methy-selenocystein [60]. How are these organic Se species released from plant materials to soil and then transformed or oxidized to soluble Se forms? Up to now, no studies have explored the specific mechanisms involved. Basidiomycota contains large numbers of fungal species that decompose organic plant material [61,62], suggesting that the Se cycle is related with the carbon cycle in soil.

Quinn *et al.* (2011) [63] conducted a litter decomposition experiment between Se hyperaccumulator plants (*Astragalus bisulcatus*) and non-accumulator species (*Astragalus drummondii* and *Medicago sativa*) in a seleniferous habitat. They found that high-Se litter supported more microbes and arthropods decomposed faster when compared to low-Se litter. Some studies have confirmed that the Se cycle processes are affected by cycles of nitrogen, carbon, sulfur, and other elements [19, 45]. Se concentration in rice grain was found to be positively connected with increasing soil nitrogen [64,65], while Li *et al.* (2015) [66] has also reported that Se content in horticulture crop and vegetable is influenced by soil nitrate. The application of nanoselenium (Se^0^) to soil enhanced SeO_4_^2−^and selenocysteine contents, and interestingly also increased soil ammonium content [56].

Many known nitrogen cycling bacterial strains are capable to reduce Se oxyanions; for example, the well documented nitrogen denitrification bacterium *Azospirillum brasilense* can reduce selenite [67]. *A. brasilense* also synthesizes extracellular selenium-sulfur nanoparticles in selenite medium under aerobic conditions [39], which implied a close and complex linkage of the Se cycle with nitrogen and sulfur cycles in soil microbial communities. The observations imply that the selenite reduction probably competes with nitrogen denitrification in *A. brasilense*. The application of phosphorus contributes to Se release from soil particles [68,69], suggesting a potential connection between phosphorus and Se cycling. While the oxidation rate by single Se-oxidizing microorganisms is quite low, the combined impacts of diverse microorganisms may be strong. Thus, the microbial communities rather than functions or activities of individual organisms are important for Se transformation in natural environments. The Se-oxidizing abilities and rates of soil microbial communities have probably been largely underestimated.

## Selenium availability in the plant–soil continuum

Understanding how plant species affect soil nutrient cycling is an important theme in different terrestrial ecosystems. Plants can directly influence the nutrient cycling via uptake, use and loss of soil nutrients [70–72]. For example, resource-acquisitive plant species with higher carbon fixation and soil nitrogen uptake induce a stronger acceleration of nitrogen cycling than resource-conservative species (slow growth) [71]. More importantly, a substantial amount of photosynthesis-derived compounds, including carbohydrates, amino acids and organic acids, show their great power in affecting soil nutrients through their influence on soil properties and recruitment of diverse microbial communities. For example, phosphatases released by roots can provide an additional source of phosphorus by hydrolyzing organic P-containing compounds [73]. The increasing P content has been shown to enhance soil Se availability [69].

The microbial community of the rhizosphere is largely determined by different plant species through sensing and responding to root-derived signals [74]. It is becoming increasingly clear that plants can control nutrient transformation mediated by diverse soil microbes in and near the rhizosphere by releasing root exudates [74–76]. An early study found a higher Se availability to rice seedlings, including soluble Se and exchangeable Se, in rhizosphere soil than in bulk soil [77], which demonstrated a potential role of plants in controlling Se transformation. As for Se transformation in soil, Oram *et al.* (2011) [78] have confirmed that a higher Se(VI) concentration was present in the rhizosphere of *Symphyotrichum eatonii* than in bulk soil, and an enhanced Se bioavailability via oxidation of reduced Se within the rhizosphere occurred.

Plants have a large impact on the physical and chemical behavior of soil due to root exudates and decomposition [79,80]. Girkin *et al.* (2018) [79] showed that low molecular weight organic acids (acetate, formate, and oxalate organic acids) can significantly increase soil pH, whereas sugars (glucose, sucrose, and fructose) decrease pH. They also found that soil redox potential increases with sugars and decreases with organic acids. Se speciation is closely related with soil properties, like pH, organic matter, and metal ion oxides, as mentioned above. Root exudates enhance the decomposition of soil organic matter and nutrient release in the rhizosphere soil [76,81]. In a review of the role of organic acids for soil Se bioavailability, Dinh *et al.* (2017) [75] summarized that many low molecular weight organic acids can increase Se bioavailability by reducing Se adsorption while promoting desorption. However, the amount and composition of plant root exudates depend largely on plant species and environments. The secretion of some organic acids, such as salicylic acid and citramalic acid, is increased by plants to solubilize phosphorus in phosphorus-deficient soil accompanied by decreasing soil pH [82]. The decreasing pH probably reduces soluble Se by promoting the adsorption of metals and organic matter to form complex compounds [32]. Plants varying in the ability of Se uptake have different influences on soil Se species, probably via root exudates. For instance, the soluble Se content in the rhizosphere soil of a Se-enriched *Oryza sativa* genotype was significantly higher than that of a non-Se-enriched rice genotype [83]. It is indicated that plants with a higher Se uptake have a stronger ability to convert insoluble Se species with a lower bioavailability to soluble Se with a high bioavailability than plants with a lower Se uptake.

Diverse microbes drive the steps of Se cycling, including reduction, oxidation, and methylation. The root exudates fuel soil microbes. Hyperaccumulators hold a greater propensity to take up and accumulate Se by transforming it into organic forms and transporting Se to root and shoot parts in seleniferous or Se-contaminated soils [70]. Se-reducing bacterial or fungal species can be recruited by plants via root exudates to immobilize selenite and selenate and to reduce these soluble anions [75,84,85]. Di Gregorio *et al.* (2006) [84] found that rhizobacteria contribute more strongly to reducing selenite and selenate oxyanions into less bioavailable forms than does the plant (*Brassica juncea*). Two fungal species, *Alternaria seleniiphila* and *Aspergillus leporis*, within the rhizosphere soil of *Stanleya pinnata* are known to reduce selenate to elemental Se [85]. *Stenotrophomonas maltophilia*, *C. testosteroni* and other rhizobacteria reduce toxic Se(IV) and Se(VI) to nontoxic Se species [85]. Transforming stable Se species in soil by different soil microbes increases the content of bioavailable Se and Se uptake by plants.

**Figure 2:**
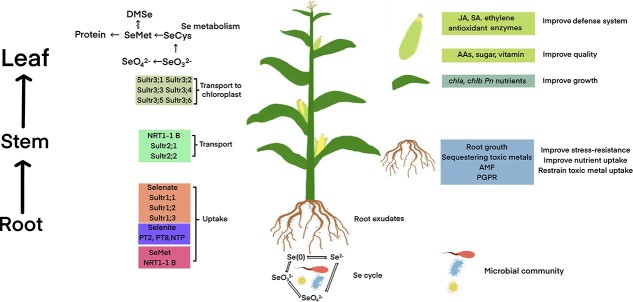
The uptake, transport, and metabolism of Se, and beneficial effects of Se on crop growth, defense, and quality. AAs: amino acids; AMF: arbuscular mycorrhizal fungi; JA: jasmonic acid; PGPR: plant growth promoting rhizobacteria; SA: salicylic acid. Se availability to crop plants depends on the balance between adsorption and desorption, reduction, methylation and oxidation, which are determined by interactions among soil properties, microbial communities and plants. Plants can affect Se fractions by root-derived compounds and by recruiting specific microbes like plant growth promoting rhizobacteria. Se is taken through transporters, including Sultr 2;1 and Sultr 2;2, and transported upward. Beneficial roles of Se in improving crop growth and quality, and enhancing protection against fungal diseases and abiotic stress.

We have reviewed the low oxidation rates by bacterial and fungal species and the interactions between microbe-driven cycling of carbon, nitrogen, and phosphorus. The abundance of AM fungi and actinomyces were found significantly positively correlated with available Se in the wheat rhizosphere soil [69]. The rhizosphere soil of the Se-enriched *O. sativa* genotype had a higher microbial biomass than that of the non-Se-enriched rice genotype [83]. The presence of AM fungi in the rhizosphere of crop roots has been verified to enhance Se bioavailability and uptake [59,69,86]. These studies imply the potential of increasing Se bioavailability by soil microbial communities recruited by plants (Fig. 2). The soil soluble Se content depends on the balance between adsorption and desorption, reduction, methylation, and oxidation as well as plant uptake. Phytoremediation, reduction, and methylation are primary pathways to reduce the soluble Se content, while Se oxidation driven by soil microbes, such as Se-oxidizing bacteria, may be weakened in Se-contaminated soils. By contrast, enhancing Se oxidation and Se desorption is crucial for the promotion of Se bioavailability and uptake, particularly in Se-deficient soils with Se supplements. Yet, the reduction and/or methylation processes would probably be impeded. However, so far, the crucial role of microbial communities in the plant–soil continuum on promoting Se bioavailability has not received much attention.

## Selenium improves crop quality and resistance to environmental stresses

The soluble selenate, selenite, and a small proportion of organic Se, such as selenomethionine, are the available forms to plants in soils (Fig. 2). The Se uptake shares a common mechanism with, e.g. sulphur and phosphorus through anion channels and transporters [64]. As having highly similar chemical traits with sulfate, selenate species are absorbed by the sulfate transporters like Sultr1;1, Sultr1;2, and Sultr1;3 [58]. The symplasmic pathway delivers most acquired selenate via the plasmodesmata to adjacent root cells, then likely being transported from roots to shoots via sulfate transporters (including Sultr 2;1 and Sultr 2;2) after being released to xylem [58]. Higher expressions of Sultr 2;1 and Sultr 2;2 lead to a greater rate of Se root-to-shoot translocation and source-to-sink remobilization [87]. The selenite uptake and transport also take place via transporters and can be affected by phosphate transporters [64,88,89]. Different studies have demonstrated that phosphate transporters OsPT2 and OsPT8 are involved in selenite uptake [64,89,90]. A silicon transporter *OsNIP2.1* in rice is also reported to increase Se uptake [64,65]. Unlike the transportation of selenate from roots to aerial parts via sulfate transporters, the majority of acquired selenite species accumulate in roots and convert into organic Se [91]. Selenomethionine in soil can enter roots via nitrate transporter NRT1.1B [58,64] and also probably via amino acid permeases located in the plasmamembrane, which mediates the movement of amino acids in a cell [92]. Then, the organic Se is transported to leaves to participate in the synthesis of Se proteins.

Beneficial roles of the Se uptake are reported in terms of improving plant growth and crop quality and enhancing protection against fungal diseases and abiotic stresses (Fig. 2) [93–95]. The edible parts of crop plants like leaves, stems, roots, seeds, fruits, and flowers are the main source of dietary Se for humans and animals [96]. After Se uptake from soil, the selenate and selenite species are mainly transformed to selenocysteine (SeCys), selenomethionine (SeMet), and methylselenocystein (MeSeCys), which then can participate in protein synthesis. Cuderman *et al.* (2008) [97] reported that 30% of total Se is used to form Se-containing proteins in potato tubers, while this proportion reaches 60–74% in golden needle mushroom (*Flammulina velutipes*) [98]. A proper dose of Se fertilizer enhances Se accumulation in different plant parts but reduces the accumulation of heavy metals [64]. The application of Se promotes concentrations of nitrogen, phosphorus, potassium, calcium, and magnesium in citrus leaves [99] and also increases potassium and calcium concentrations of grape berries [100]. The nitrogen, potassium, and calcium uptake of wheat seedlings (*Triticum aestivum* L.) was enhanced by the application of 5 μM Na_2_SeO_4_ but the nitrogen uptake was significantly declined by10 μM Na_2_SeO_4_ [101]. Drahoňovský *et al.* (2016) [102] investigated the effects of selenate on the uptake of many soil elements in 12 plant species and suggested that the improved nutrient uptake largely depends on the crop species and its growth environment.

Many studies have reported that Se fertilizer promotes the chlorophyll and photosynthesis rate [99,100,103]. The improved photosynthetic traits can supply sufficient carbon for the growth of roots, shoots and other edible parts [98,104]. For example, a 150 mg/L Na_2_SeO_3_ application increased the size and weight, and contents of total sugar and vitamins, but decreased the fruit acidity and the pericarp thickness of citrus fruit (*Citrus reticulata* Blanco cv. Succosa) [99]. Similarly, the grape berries showed an increase in sugars, vitamin C and soluble proteins, but a decline in organic acids after a proper Se fertilizer was applied [100]. In addition, the grape berries showed an increased acid invertase activity, which plays an important role in sugar accumulation and could explain the increased soluble sugar content [105]. The carbohydrate content of rice grain is also promoted by a Se fertilizer [64]. Sugar transporter genes of tea plants (*Camellia sinensis*) were upregulated in response to a Se application [106]. All these results suggest a positive effect of proper Se on carbohydrate accumulation, which involves carbon fixation, transport, and metabolism.

Like nitrogen, potassium, and calcium, the Se metabolism of plants is closely associated with the metabolism of nitrogenous substances, especially with amino acids. Some studies have reported an increase in protein contents, such as those in rice grain, potato, and grape [64,100]. Ježek *et al.* (2011) [107] explored responses of 17 amino acids in potato tubers to Se application and reported significantly increased levels of phenylalanine, aspartic acid, glutamic acid, threonine, and tyrosine. Phenylalanine is reported to have an average increase of 46% [107]. Similarly, the increased Se level promotes protein and total amino acid contents of *F. velutipes*, indicating an increased nutritional quality [98]. In some crops, Se could be used as a regulator to manipulate the concentrations of amino acids that are essential for the quality. For example, the amount of amino acids is one of the most important traits in estimating the flavor and quality of tea, because amino acids supply umami and sweet taste and, thus, unique aroma to tea. An enhanced synthesis of diverse amino acids, including L-phenylalanine, L-lysine acid, L-glutamate, L-arginine, and increased epigallocatechin and epigallocatechin gallate contents (contributing to the tea quality) after Se application demonstrate a great improvement in the tea quality [108]. Li *et al.* (2021) [109] have reported a marked increase in theanine, glutamic acid, proline, and arginine by adjusting the GS-GOGAT cycle under Se application, and Se also promotes gene expression related to amino acid and protein metabolism in tea plants [106]. The enhanced nitrogen metabolism may be primarily driven by the increased nitrogen uptake under Se fertilization. However, the specific mechanisms of how Se promotes nitrogen uptake is still lacking.

Crop species are vulnerable to various abiotic and biotic stresses, such as drought, heavy metals, herbivory, and diseases. Besides improving crop quality and plant growth, Se is a protective element for plants against toxic metals and pathogenic microbes, as explored in many studies [110–112]. Gui *et al.* (2022) [113] and Lai *et al.* (2022) [114] have summarized the crucial roles of Se in reducing metal toxicity to plants, for example, by reducing the mobility of metal ions by alternating soil pH and inhibiting upward transport by sequestering toxic metals inside the vacuoles. Many studies have reported that Se promotes plants’ defense systems when facing diverse stresses [111,115]. Many amino acids play crucial roles in defense systems against stresses. For example, phenylpropanoids with significant antioxidant effects are derived from phenylalanine and tyrosine, which are promoted by Se fertilization in potato tubers [107]. Defense-related genes associated with the synthesis and signaling of jasmonic acid, salicylic acid and ethylene are more strongly expressed in *S. pinnata* (Se hyperaccumulator) than in *Stanleya elata* (nonaccumulator) when growing in a medium with 20 μM selenate [116]. Alleviating oxidative stress and regulating the activity of antioxidant enzymes are crucial Se-protective responses of crop plants to various stresses. Many studies have reported that exogenous Se application greatly reduces reactive oxygen species produced by crops exposed to stresses [95,115]. For example, by inducing disproportionation of O_2_^.-^ to produce H_2_O_2_, which is then decomposed under antioxidant enzymes, mediating reduction in electrolytic leakage with improved cell integrity, Se can inhibit oxidative damage of plant cells [117,118].

The metabolism of Se increases antioxidant enzyme activities, including superoxide dismutase, peroxidase, and catalase, which further reduces oxidative damage caused by stresses [19,94–108]. Se can facilitate the biosynthesis of pigments, such as chlorophyll, by improving nutrient accumulation (e.g., Fe), thus being beneficial for the photosynthetic system (Fig. 2) [94,119]. SeCys constitutes the active glumathione peroxidase center, which catalyzes the synthesis of glutathione. While glutathione combines with ascorbic acid to decompose H_2_O_2_, it is also involved in the synthesis of phytochelatins with a high ability of sequestering toxic metals [120]. Many studies have shown the effects of Se on heavy metal detoxification and accumulation reduction [94,95]. Most heavy metals can bind Se species to form complex compounds that cannot be absorbed by plants, thus indirectly reducing the toxicity of heavy metals [56,95,121]. Crop plants are healthier to humans and animals when Se reduces the accumulation of heavy metals.

Patel *et al.* (2018) [122] have suggested the enhancement of Se concentration via microorganisms (*Selenorhizobacteria*) as sustainable biotechnological tools to protect plants against stresses and to increase plants’ nutrition and quality. Besides improving plants’ defense systems, applying Se can help plants to recruit and boost beneficial microorganisms in the rhizosphere soil to protect them further [56]. For instance, many beneficial microbial species belonging to *Gammaproteobacteria*, *Alphaproteobacteria*, *Bacteroidia*, *Gemmatimonadetes*, *Deltaproteobacteria*, and *Anaerolineae* are enriched in the rhizosphere soil of pepper (*Capsicum annuum* L.) to protect against Cd stress after an exogenous Se application [56]. Root secretion fuels diverse microorganisms and shapes their communities [62,123]. It will be interesting to find out the role of Se in affecting soil microbial communities via altering root exudates.

Endophytes colonizing the interior of any plant part (such as roots, stems, and leaves), can improve plant growth and protect plants against environmental stresses [124–127]. It was recently discovered that a fungal endophyte *Alternaria tenuissima* isolated from the hyperaccumulator plant *A. bisulcatus* can transform Se species to Se(0) or methylated organic Se in the host, thereby removing Se toxicity to the host. This suggests the potential of endophytes to affect plant properties relevant for phytoremediation [125]. A tea plant-specific endophytic bacterium, *Herbaspirillum* sp. strain WT00C markedly enhanced Se enrichment [127], while it was reported to be capable of producing indole-3-acetic acid, ammonia and siderophores to improve plant growth [128]. Endophytic selenobacteria, such as *Alcaligenes faecalis* and *S. maltophilia*, and especially *Paraburkholderia megapolitana*, greatly improve the growth of *Glycine max* under drought because of exogenous Se application [126]. The endophytes successfully colonize plant roots and leaves probably through overcoming or escaping the plants’ immune system [129]. Some studies suggest that the metabolism and immune-related traits of plants are key factors in regulating or selecting a specific microbiota that contribute to stress resistance [129,130]. The Se application largely influences plants’ physiological and transcriptome traits [131,132], and probably imposes selective pressure to form specific endophytic communities related with stress resistance.

## Conclusions and perspectives

In summary, the impact of soil properties, microorganisms and plant genotype on Se speciation and transformation along the soil–plant continuum has received increasing attention during recent years. This review highlighted the crucial roles of microorganisms and plants in driving the biochemical cycle of Se. The microbial isolates causing Se reduction and methylation rapidly immobilize or volatilize soluble Se species. These processes are important in reducing potential risks of highly soluble Se for non-hyperaccumulator plant species. These isolates are suggested to be recruited and enriched by plant root secretion in seleniferous and Se-contaminated environments. Plants recruit growth promoting microorganisms in soil and harbor diverse endophytes, including AM fungi. These microorganisms enhance Se absorption and accumulation, which, in turn, increase plant quality and stress resistance. Therefore, it is important to enhance Se accumulation in crop plants by increasing soil Se availability (e.g. via Se-oxidizing bacteria) in Se-limited areas.

Current understanding of the biochemical cycle pathways of Se relies on culture-based approaches, i.e. isolating the individual microorganism involved in Se species transformation. However, the pathways of the whole biochemical cycle of Se are poorly understood, although they have been shown to be closely connected with carbon, N, P, and S cycles. One of the main obstacles is that the functional genes involved in Se transformation have rarely been studied. It would be necessary to investigate how soil microbial communities differing in composition and function affect gene functions related to the Se cycle. Furthermore, there is a pressing need to explore specific mechanisms of plants that affect Se bioavailability by harboring different microbial communities. Nowadays, appropriate concentrations of exogenous Se are applied in Se-limited areas to increase crop yield, and to improve crop quality and stress tolerance. Understanding Se speciation and transformation along the plant–soil continuum is crucial for agricultural production and even for human health.

## Data Availability

No new data was generated for the research reviewed in the article.

## References

[1] Santi C , Bagnoli L. Celebrating two centuries of research in selenium chemistry: state of the art and new prospective. *Molecules.* 2017;22:2124. 29207462 10.3390/molecules22122124 PMC6149956

[2] Ekumah JN , Ma Y, Akpabli-Tsigbe NDK et al. Global soil distribution, dietary access routes, bioconversion mechanisms and the human health significance of selenium: a review. *Food Biosci.* 2021;41:100960.

[3] Vriens B , Lenz M, Charlet L et al. Natural wetland emissions of methylated trace elements. *Nat Commun.* 2014;5:3035. 24398909 10.1038/ncomms4035

[4] Li S , Bañuelos GS, Wu L et al. The changing selenium nutritional status of Chinese residents. *Nutrients.* 2014;6:1103–14. 24638069 10.3390/nu6031103 PMC3967180

[5] Fordyce FM . Selenium deficiency and toxicity in the environment. In: Essentials of Medical Geology. Springer, 2013,375–416.

[6] WHO . Vitamin and Mineral Requirements in Human Nutrition. World Health Organization; 2004.

[7] Winkel LHE , Johnson CA, Lenz M et al. Environmental selenium research: from microscopic processes to global understanding. *Environ Sci Technol.* 2012;46:571–9. 22129299 10.1021/es203434d

[8] Wen H , Carignan J. Reviews on atmospheric selenium: emissions, speciation and fate. *Atmos Environ.* 2007;41:7151–65.

[9] Khoei NS , Lampis S, Zonaro E et al. Insights into selenite reduction and biogenesis of elemental selenium nanoparticles by two environmental isolates of *Burkholderia fungorum*. *New Biotechnol.* 2017;34:1–11. 10.1016/j.nbt.2016.10.002 27717878

[10] Rosenfeld CE , Kenyon JA, James BR et al. Selenium (IV, VI) reduction and tolerance by fungi in an oxic environment. *Geobiology.* 2017;15:441–52. 28044397 10.1111/gbi.12224

[11] Goff J , Terry L, Mal J et al. Role of extracellular reactive sulfur metabolites on microbial se(0) dissolution. *Geobiology.* 2019;17:320–9. 30592130 10.1111/gbi.12328

[12] Xu WF , Chen QX, Shi WM. Effects of nitrate supply site on selenite uptake by rice roots. *J Agric Food Chem.* 2010;58:11075–80. 20923148 10.1021/jf102263e

[13] Zhu YG , Pilon-Smits EAH, Zhao FJ et al. Selenium in higher plants: understanding mechanisms for biofortification and phytoremediation. *Trends Plant Sci.* 2009;14:436–42. 19665422 10.1016/j.tplants.2009.06.006

[14] Thiry C , Ruttens A, De Temmerman L et al. Current knowledge in species-related bioavailability of selenium in food. *Food Chem.* 2012;130:767–84.

[15] Ali F , Peng Q, Wang D et al. Effects of selenite and selenate application on distribution and transformation of selenium fractions in soil and its bioavailability for wheat (*Triticum aestivum* L.). *Environ Sci Pollut R.* 2017;24:8315–25. 10.1007/s11356-017-8512-9 28161863

[16] Dinh DT , Wang M, Tran TAT et al. Bioavailability of selenium in soil-plant system and a regulatory approach. *Crit Rev Env Sci Tec.* 2019;49:443–517.

[17] Wang C , Ji J, Zhu F. Characterizing se transfer in the soil-crop systems under field condition. *Plant Soil.* 2017;415:535–48.

[18] Weng L , Vega FA, Supriatin S et al. Speciation of se and DOC in soil solution and their relation to se bioavailability. *Environ Sci Technol.* 2011;45:262–7. 21141820 10.1021/es1016119

[19] Wang D , Rensing C, Zheng S. Microbial reduction and resistance to selenium: mechanisms, applications and prospects. *J Hazard Mater.* 2022;421:126684. 34339989 10.1016/j.jhazmat.2021.126684

[20] Lyu C , Qin Y, Zhao Z et al. Characteristics of selenium enrichment and assessment of selenium bioavailability using the diffusive gradients in thin-films technique in seleniferous soils in Enshi, Central China. *Environ Pollut.* 2021;273:116507. 33493758 10.1016/j.envpol.2021.116507

[21] Bolan N , Adriano D, Mahimairaja S. Distribution and bioavailability of trace elements in livestock and poultry manure by-products. *Crit Rev Env Sci Tec.* 2004;34:291–338.

[22] Bassil J , Naveau A, Bueno M et al. Leaching behavior of selenium from the karst infillings of the hydrogeological experimental site of Poitiers. *Chem Geol.* 2018;483:141–50. 10.1016/j.chemosphere.2021.129935 33667770

[23] Dhillon K , Dhillon S. Adsorption-desorption reactions of selenium in some soils of India. *Geoderma.* 1999;93:19–31.

[24] Nakamaru YM , Altansuvd J. Speciation and bioavailability of selenium and antimony in non-flooded and wetland soils: a review. *Chemosphere.* 2014;111:366–71. 24997941 10.1016/j.chemosphere.2014.04.024

[25] Natasha SM , Niazi NK, Khalid S et al. A critical review of selenium biogeochemical behavior in soil-plant system with an inference to human health. *Environ Pollut.* 2018;234:915–34. 29253832 10.1016/j.envpol.2017.12.019

[26] Peak D , Sparks DL. Mechanisms of selenate adsorption on iron oxides and hydroxides. *Environ Sci Technol.* 2002;36:1460–6. 11999051 10.1021/es0156643

[27] Peak D . Adsorption mechanisms of selenium oxyanions at the aluminum oxide/water interface. *J Colloid Interf Sci.* 2006;303:337–45. 10.1016/j.jcis.2006.08.014 16949599

[28] Shaheen SM , Frohne T, White JR et al. Redox-induced mobilization of copper, selenium, and zinc in deltaic soils originating from Mississippi (USA) and Nile (Egypt) river deltas: a better understanding of biogeochemical processes for safe environmental management. *J Environ Manag.* 2017;186:131–40. 10.1016/j.jenvman.2016.05.032 27240716

[29] Liu Y , Tian X, Liu R et al. Key driving factors of selenium-enriched soil in the low-se geological belt: a case study in red beds of Sichuan Basin, China. *Catena.* 2021a;196:104926.

[30] Twidwell L . The removal of arsenic, selenium and metals from aqueous solution by iron precipitation and reduction techniques. In: TMS2011 Annual Meeting. San Diego, CA, 2011,104926.

[31] Qin HB , Zhu JM, Su H. Selenium fractions in organic matter from se-rich soils and weathered stone coal in selenosis areas of China. *Chemosphere.* 2012;86:626–33. 22115469 10.1016/j.chemosphere.2011.10.055

[32] Zhai H , Kleawsampanjai P, Wang M et al. Effects of soil moisture on aging of exogenous selenate in three different soils and mechanisms. *Geoderma.* 2021;390:114966.

[33] Pinsent J . The need for selenite and molybdate in the formation of formic dehydrogenase by members of the *coli-aerogenes* group of bacteria. *Biochem J.* 1954;57:10–6. 13159942 10.1042/bj0570010 PMC1269698

[34] Acuña JJ , Jorquera MA, Barra PJ et al. Selenobacteria selected from the rhizosphere as a potential tool for se biofortification of wheat crops. *Biol Fert Soils.* 2013;49:175–85.

[35] Huang CL , Wang HL, Shi XY et al. Two new selenite reducing bacterial isolates from paddy soil and the potential se biofortification of paddy rice. *Ecotoxicology.* 2021;30:1465–75. 32880083 10.1007/s10646-020-02273-6

[36] Oremland RS , Steinberg NA, Presser TS et al. In situ bacterial selenate reduction in the agricultural drainage systems of western Nevada. *Appl Environ Microb.* 1991;57:615–7. 10.1128/aem.57.2.615-617.1991 PMC182762 2014995

[37] Pinel-Cabello M , Chapon V, Ruiz-Fresneda MA et al. Delineation of cellular stages and identification of key proteins for reduction and biotransformation of se(IV) by *Stenotrophomonas bentonitica* BII-R7. *J Hazard Mate.* 2021;418:126150. 10.1016/j.jhazmat.2021.126150 34111750

[38] Nancharaiah YV , Lens P. Ecology and biotechnology of selenium-respiring bacteria. *Microbiol Mo Biol R.* 2015;79:61–80. 10.1128/MMBR.00037-14 PMC4402961 25631289

[39] Eswayah AS , Hondow N, Scheinost AC et al. Methyl selenol as a precursor in selenite reduction to se/S species by methane-oxidizing bacteria. *Appl Environ Microb.* 2019;85:e01379–19. 10.1128/AEM.01379-19 PMC6821961 31519658

[40] Wells M , Stolz JF. Microbial selenium metabolism: a brief history, biogeochemistry and ecophysiology. *FEMS*. *Microb Ecol.* 2020;96:fiaa209. 10.1093/femsec/fiaa209 33045045

[41] Bacon M , Ingledew WJ. The reductive reactions of *Thiobacillus ferrooxidans* on Sulphur and selenium. *FEMS Microbiol Lett.* 1989;58:189–94.

[42] Herbel MJ , Blum JS, Oremland RS et al. Reduction of elemental selenium to selenide: experiments with anoxic sediments and bacteria that respire se-oxyanions. *Geomicrobiology.* 2003;20:587–602.

[43] Zehr JP , Oremland RS. Reduction of selenate to selenide by sulfate-respiring bacteria: experiments with cell suspensions and estuarine sediments. *Appl Environ Microb.* 1987;53:1365–9. 10.1128/aem.53.6.1365-1369.1987 PMC203871 16347366

[44] Zambonino MC , Quizhpe EM, Jaramillo FE et al. Green synthesis of selenium and tellurium nanoparticles: current trends, biological properties and biomedical applications. *Int J Mol Sci.* 2021;22:989. 33498184 10.3390/ijms22030989 PMC7863925

[45] Rosenfeld CE , Sabuda MC, Hinkle MAG et al. A fungal-mediated cryptic selenium cycle linked to manganese biogeochemistry. *Environ Sci Technol.* 2020;54:3570–80. 32083848 10.1021/acs.est.9b06022

[46] Liang X , Perez M, Nwoko KC et al. Fungal formation of selenium and tellurium nanoparticles. *Appl Microbiol Biot.* 2019;103:7241–59. 10.1007/s00253-019-09995-6 PMC6691031 31324941

[47] Liang X , Perez MJ, Zhang S et al. Fungal transformation of selenium and tellurium located in a volcanogenic sulfide deposit. *Environ Microbiol.* 2020;22:2346–64. 32250010 10.1111/1462-2920.15012

[48] Torma AE , Habashi F. Oxidation of copper (II) selenide by *Thiobacillus ferrooxidans*. *Can J Microbiol* 1972;18:1780–1. 5086113 10.1139/m72-278

[49] Sarathchandra S , Watkinson J. Oxidation of elemental selenium to selenite by *bacillus megaterium*. *Science.* 1981;211:600–1. 6779378 10.1126/science.6779378

[50] Losi ME , Frankenberger WT. Microbial oxidation and solubilization of precipitated elemental selenium in soil. *J Environ Qual.* 1998;27:836–43.

[51] Dinh DT , Cui Z, Huang J et al. Selenium distribution in the Chinese environment and its relationship with human health: a review. *Environ Int.* 2018;112:294–309. 29438838 10.1016/j.envint.2017.12.035

[52] Wang D , Dinh QT, Thu TTA et al. Effect of selenium-enriched organic material amendment on selenium fraction transformation and bioavailability in soil. *Chemosphere.* 2018a;199:417–26. 29453068 10.1016/j.chemosphere.2018.02.007

[53] Luo X , Wang YT, Lan Y et al. Microbial oxidation of organic and elemental selenium to selenite. *Sci Total Environ.* 2022;833:155203. 35421462 10.1016/j.scitotenv.2022.155203

[54] Zhu D , Niu Y, Fan K et al. Selenium-oxidizing *agrobacterium* sp. T3F4 steadily colonizes in soil promoting selenium uptake by pak choi (*Brassica campestris*). *Sci Total E nviron.* 2021;791:148294. 10.1016/j.scitotenv.2021.148294 34126490

[55] Dowdle PR , Oremland RS. Microbial oxidation of elemental selenium in soil slurries and bacterial cultures. Environ. Sci. Technol. 1998;32: 3749-3755.

[56] Li D , Zhou C, Wu Y et al. Nanoselenium integrates soil-pepper plant homeostasis by recruiting rhizosphere-beneficial microbiomes and allocating signaling molecule levels under cd stress. *J Hazard Mater.* 2022a;432:128763. 35349848 10.1016/j.jhazmat.2022.128763

[57] Zhang L , Zhou J, George TS et al. Arbuscular mycorrhizal fungi conducting the hyphosphere bacterial orchestra. *Trends Plant Sci.* 2021;27:402–11. 34782247 10.1016/j.tplants.2021.10.008

[58] Zhang L , Chu C. Selenium uptake, transport, metabolism, reutilization, and biofortification in rice. *Rice.* 2022;15:30. 35701545 10.1186/s12284-022-00572-6 PMC9198118

[59] Chen X , Zhang Z, Gu M et al. Combined use of arbuscular mycorrhizal fungus and selenium fertilizer shapes microbial community structure and enhances organic selenium accumulation in rice grain. *Sci Total Environ.* 2020;748:141166. 32798860 10.1016/j.scitotenv.2020.141166

[60] Wang M , Ali F, Qi M et al. Insights into uptake, accumulation, and subcellular distribution of selenium among eight wheat (*Triticum aestivum* L.) cultivars supplied with selenite and selenate. *Ecotox Environ Safe.* 2021;207:111544. 10.1016/j.ecoenv.2020.111544 33254403

[61] Baldrian P . Fungal laccases – occurrence and properties. *FEMS Microbiol Revi.* 2006;30:215–42. 10.1111/j.1574-4976.2005.00010.x 16472305

[62] Guo Q , Yan L, Korpelainen H et al. Plant-plant interactions and N fertilization shape soil bacterial and fungal communities. *Soil Biol Biochem.* 2019;128:127–38.

[63] Quinn CF , Wyant KA, Wangeline AL et al. Enhanced decomposition of selenium hyperaccumulator litter in a seleniferous habitat-evidence for specialist decomposers? *Plant Soil.* 2011;341:51–61.

[64] Teixeira LS , Pimenta TM, Brito FAL et al. Selenium uptake and grain nutritional quality are affected by nitrogen fertilization in rice (*Oryza sativa L*.). *Plant Cell Rep.* 2021;40:871–80. 33772600 10.1007/s00299-021-02685-6

[65] Zhao X , Mitani N, Yamaji N et al. Involvement of silicon influx transporter OsNIP2;1 in selenite uptake in rice. *Plant Physiol.* 2010;153:1871–7. 20498338 10.1104/pp.110.157867 PMC2923891

[66] Li S , Bañuelos GS, Min J et al. Effect of continuous application of inorganic nitrogen fertilizer on selenium concentration in vegetable grown in the Taihu Lake region of China. *Plant Soil.* 2015;393:351–60.

[67] Tugarova AV , Vetchinkina EP, Loshchinina EA et al. Reduction of selenite by *Azospirillum brasilense* with the formation of selenium nanoparticles. *Microb Ecol.* 2014;68:495–503. 24863127 10.1007/s00248-014-0429-y

[68] Peng Q , Wu M, Zhang Z et al. The interaction of Arbuscular Mycorrhizal fungi and phosphorus inputs on selenium uptake by alfalfa (Medicago sativa L.) and selenium fraction transformation in soil. *Front Plant Sci.* 2020;11:966. 32676094 10.3389/fpls.2020.00966 PMC7333729

[69] Li J , Liu R, Zhang C et al. Selenium uptake and accumulation in winter wheat as affected by level of phosphate application and arbuscular mycorrhizal fungi. *J Hazard Mater.* 2022b;433:128762. 35358814 10.1016/j.jhazmat.2022.128762

[70] Jason R , Reynolds B, Pilon-Smits EAH. Plant selenium hyperaccumulation-ecological effects and potential implications for selenium cycling and community structure. *BBA-Gen Subjects.* 2018;1862:2372–82. 10.1016/j.bbagen.2018.04.018 29704528

[71] Henneron L , Kardol P, Wardle DA et al. Rhizosphere control of soil nitrogen cycling: a key component of plant economic strategies. *New Phytol.* 2020;228:1269–82. 32562506 10.1111/nph.16760

[72] Guo Q , Liu J, Yu L et al. Different sexual impacts of dioecious *Populus euphratica* on microbial communities and nitrogen cycle processes in natural forests. *Forest Ecol Manag.* 2021;496:119403.

[73] Richardson AE , Lynch JP, Ryan PR et al. Plant and microbial strategies to improve the phosphorus efficiency of agriculture. *Plant Soil.* 2011;349:121–56.

[74] Mushinski RM , Payne ZC, Raff JD et al. Nitrogen cycling microbiomes are structured by plant mycorrhizal associations with consequences for nitrogen oxide fluxes in forests. *Glob Chang Biol.* 2021;27:1068–82. 10.1111/gcb.15439 PMC7898693 33319480

[75] Dinh DT , Li Z, Tran TAT et al. Role of organic acids on the bioavailability of selenium in soil: a review. *Chemosphere.* 2017;184:618–35. 28624740 10.1016/j.chemosphere.2017.06.034

[76] Liu Y , Evans SE, Friesen ML et al. Root exudates shift how N mineralization and N fixation contribute to the plant-available N supply in low fertility soils. *Soil Biol Biochem.* 2022;165:108541.

[77] Chen QX , Shi WM, Wang XC. Selenium speciation and distribution characteristics in the rhizosphere soil of rice (*Oryza sativa* L.) seedlings. *Commun Soil Sci Plant Anal.* 2010;41:1411–25.

[78] Oram LL , Strawn DG, Möller G. Chemical speciation and bioavailability of selenium in the rhizosphere of *Symphyotrichum eatonii* from reclaimed mine soils. *Environ Sci Technol.* 2011;45:870–5. 21166454 10.1021/es1029766

[79] Girkin NT , Turner BL, Ostle N et al. Composition and concentration of root exudate analogues regulate greenhouse gas fluxes from tropical peat. *Soil Biol Biochem* 2018;127:280–5.

[80] Oleghe E , Naveed M, Baggs EM et al. Residues with varying decomposability interact differently with seed or root exudate compounds to affect the biophysical behaviour of soil. *Geoderma.* 2019;343:50–9.

[81] Haichar FE , Santaella C, Heulin T et al. Root exudates mediated interactions belowground. *Soil Biol Biochem.* 2014;77:69–80.

[82] Khorassani R , Hettwer U, Ratzinger A et al. Citramalic acid and salicylic acid in sugar beet root exudates solubilize soil phosphorus. *BMC Plant Biol.* 2011;11:121. 21871058 10.1186/1471-2229-11-121 PMC3176199

[83] Zhou XB , Zhang CM, Gao AX. Selenium speciation and distribution in the rhizosphere and selenium uptake of two rice (*Oryza sativa*) genotypes. *Int J Agric Biol.* 2018;20:136–42.

[84] Di Gregorio S , Lampis S, Malorgio F et al. Brassica juncea can improve selenite and selenate abatement in selenium contaminated soils through the aid of its rhizospheric bacterial population. *Plant Soil.* 2006;285:233–44.

[85] Lindblom SD , Fakra SC, Landon J et al. Inoculation of selenium hyperaccumulator *Stanleya pinnata* and related non-accumulator *Stanleya elata* with hyperaccumulator rhizosphere fungi – investigation of effects on se accumulation and speciation. *Physiol Plant.* 2014;150:107–18. 24032473 10.1111/ppl.12094

[86] Larsen EH , Lobinski R, Burger-Meyer K et al. Uptake and speciation of selenium in garlic cultivated in soil amended with symbiotic fungi (mycorrhiza) and selenate. *Anal Bioanal Chem.* 2006;385:1098–108. 16770577 10.1007/s00216-006-0535-x

[87] Cappa JJ , Cappa PJ, El Mehdawi AF et al. Characterization of selenium and sulfur accumulation across the genus *Stanleya* (Brassicaceae): a field survey and common-garden experiment. *Am J Bot.* 2014;101:830–9. 24752889 10.3732/ajb.1400041

[88] Li HF , McGrath SP, Zhao FJ. Selenium uptake, translocation and speciation in wheat supplied with selenate or selenite. *New Phytol.* 2008;178:92–102. 18179602 10.1111/j.1469-8137.2007.02343.x

[89] Zhang L , Hu B, Li W et al. OsPT2, a phosphate transporter, is involved in the active uptake of selenite in rice. *New Phytol.* 2014a;201:1191. 10.1111/nph.12596 PMC4284032 24491113

[90] Song Z , Shao H, Huang H et al. Overexpression of the phosphate transporter gene OsPT8 improves the pi and selenium contents in *Nicotiana tabacum*. *Environ Exp Bot.* 2017;137:158–65.

[91] Zhang L , Hu B, Deng K et al. NRT1.1B improves selenium concentrations in rice grains by facilitating selenomethinone translocation. *Plant Biotechnol J.* 2019;17:1058–68. 30466149 10.1111/pbi.13037 PMC6523590

[92] White PJ , Broadley MR. Biofortification of crops with seven mineral elements often lacking in human diets - iron, zinc, copper, calcium, magnesium, selenium and iodine. *New Phytol.* 2009;182:49–84. 19192191 10.1111/j.1469-8137.2008.02738.x

[93] Hanson B , Garifullina GF, Lindblom SD et al. Selenium accumulation protects *Brassica juncea* from invertebrate herbivory and fungal infection. *New Phytol.* 2003;159:461–9. 33873368 10.1046/j.1469-8137.2003.00786.x

[94] Ulhassan Z , Gill RA, Huang H et al. Selenium mitigates the chromium toxicity in *Brassicca napus* L. by ameliorating nutrients uptake, amino acids metabolism and antioxidant defense system. *Plant Physiol Biochem.* 2019;145:142–52. 31689666 10.1016/j.plaphy.2019.10.035

[95] Zhou XB , Yang J, Kronzucher HJ et al. Selenium biofortification and interaction with other elements in plants: a review. *Front Plant Sci.* 2020;11:586421. 33224171 10.3389/fpls.2020.586421 PMC7674621

[96] Yang XY , Liao XL, Yu L et al. Combined metabolome and transcriptome analysis reveal the mechanism of selenate influence on the growth and quality of cabbage (*Brassica oleracea* var. *capitata* L.). *Food Res Int.* 2022;156:111135. 35651008 10.1016/j.foodres.2022.111135

[97] Cuderman P , Kreft I, Germ M et al. Selenium species in selenium-enriched and drought-exposed potatoes. *J Agr Food Chem.* 2008;56:9114–20. 18795781 10.1021/jf8014969

[98] Dong Z , Xiao Y, Wu H. Selenium accumulation, speciation, and its effect on nutritive value of *Flammulina velutipes* (Golden needle mushroom). *Food Chem.* 2021;350:128667. 33288349 10.1016/j.foodchem.2020.128667

[99] Wen M , Wang P, Gao W et al. Effects of foliar spraying with different concentrations of selenium fertilizer on the development, nutrient absorption, and quality of citrus fruits. *HortScience.* 2021;56:1363–7.

[100] Zhu S , Liang Y, Gao D et al. Spraying foliar selenium fertilizer on quality of table grape (*Vitis vinifera* L*.*) from different source varieties. *Sci Hortic.* 2017a;218:87–94.

[101] Elkelish AA , Soliman MH, Alhaithloul HA et al. Selenium protects wheat seedlings against salt stress-mediated oxidative damage by up-regulating antioxidants and osmolytes metabolism. *Plant Physiol Biochem.* 2019;137:144–53. 30784986 10.1016/j.plaphy.2019.02.004

[102] Drahoňovský J , Száková J, Mestek O et al. Selenium uptake, transformation and inter-element interactions by selected wildlife plant species after foliar selenate application. *Environ Exp Bot.* 2016;125:12–9.

[103] Mozafariyan M , Pessarakli M, Saghafi K. Effects of selenium on some morphological and physiological traits of tomato plants grown under hydroponic condition. *J Plant Nutr.* 2017;40:139–44.

[104] Alves LR , Rossatto DR, Rossi ML et al. Selenium improves photosynthesis and induces ultrastructural changes but dose not alleviate cadmium-stress damages in tomato plants. *Protoplasma.* 2020;257:597–605. 31844994 10.1007/s00709-019-01469-w

[105] Zhu S , Liang Y, An X et al. Changes in sugar content and related enzyme activities in table grape (*Vitis vinifera* L.) in response to foliar selenium fertilizer. *J Sci Food Agr.* 2017b;97:4094–102. 28211621 10.1002/jsfa.8276

[106] Ren HZ , Li XM, Guo LN et al. Integrative Transcriptome and proteome analysis reveals the absorption and metabolism of selenium in tea plants [Camellia sinensis (L.) O. Kuntze]. *Front Plant Sci.* 2022;13:848349. 35283867 10.3389/fpls.2022.848349 PMC8908381

[107] Ježek P , Hlušek J, Lošák T et al. Effect of foliar application of selenium on the content of selected amino acids in potato tubers (*Solanum tuberosum* L.). *Plant Soil Environ.* 2011;57:315–20.

[108] Liu K , Li S, Han J et al. Effect of selenium on tea (*Camellia sinensis*) under low temperature: changes in physiological and biochemical responses and quality. *Environ Exp Bot.* 2021b;188:104475.

[109] Li D , Zhou CR, Zou N et al. Nanoselenium foliar application enhances biosynthesis of tea leaves in metabolic cycles and associated responsive pathways. *Environ Pollut.* 2021;273:116503. 33486255 10.1016/j.envpol.2021.116503

[110] Sae-Lee N , Kerdchoechuen O, Laohakunjit N. Chemical qualities and phenolic compounds of Assam tea after soil drench application of selenium and aluminium. *Plant Sci.* 2012;356:381–93.

[111] Wu C , Dun Y, Zhang Z et al. Foliar application of selenium and zinc to alleviate wheat (*Triticum aestivum* L.) cadmium toxicity and uptake from cadmium-contaminated soil. *Ecotox Environ Safe.* 2020;190:110091. 10.1016/j.ecoenv.2019.110091 31881404

[112] Troni E , Beccari G, D'Amato R et al. In vitro evaluation of the inhibitory activity of different selenium chemical forms on the growth of a *Fusarium proliferatum* strain isolated from rice seedlings. *Plants-Basel.* 2021;10:1725. 34451770 10.3390/plants10081725 PMC8398910

[113] Gui JY , Rao S, Huang XR et al. Interaction between selenium and essential micronutrient elements in plants: a systematic review. *Sci Total Environ.* 2022;853:158673. 36096215 10.1016/j.scitotenv.2022.158673

[114] Lai X , Yang X, Rao S et al. Advances in physiological mechanisms of selenium to improve heavy metal stress tolerance in plants. *Plant Biol J.* 2022;24:913–9. 10.1111/plb.13435 35583793

[115] Jiang H , Lin W, Jiao H et al. Uptake, transport, and metabolism of selenium and its protective effects against toxic metals in plants: a review. *Metallomics.* 2021;13:mfab040. 34180517 10.1093/mtomcs/mfab040

[116] Wang J , Cappa JJ, Harris JP et al. Transcriptome-wide comparison of selenium hyperaccumulator and nonaccumulator *Stanleya* species provides new insight into key processes mediating the hyperaccumulation syndrome. *Plant Biotechnol J.* 2018b;16:1582–94. 29412503 10.1111/pbi.12897 PMC6097121

[117] Malik JA , Goel S, Kaur N et al. Selenium antagonizes the toxic effects of arsenic on mungbean (*Phaseolus aureus* Roxb.) plants by restricting its uptake and enhancing the antioxidative and detoxification mechanisms. *Environ Exp Bot.* 2012;77:242–8.

[118] Zwolak I . The role of selenium in arsenic and cadmium toxicity: an updated review of scientific literature. *Biol Trace Elem Res.* 2020;193:44–63. 30877523 10.1007/s12011-019-01691-w PMC6914719

[119] Leticia Rodrigues A , Davi Rodrigo R, Mnica Lanzoni R et al. Selenium improves photosynthesis and induces ultrastructural changes but does not alleviate cadmium-stress damages in tomato plants. *Protoplasma.* 2020;257:597–605. 31844994 10.1007/s00709-019-01469-w

[120] Sharma SS , Dietz KJ. The significance of amino acids and amino acid-derived molecules in plant responses and adaptation to heavy metal stress. *J Exp Bot.* 2006;57:711–26. 16473893 10.1093/jxb/erj073

[121] Zhao J , Liang X, Zhu N et al. Immobilization of mercury by nano-elemental selenium and the underlying mechanisms in hydroponic-cultured garlic plant. *Environ Sci-Nano.* 2020;7:1115–25.

[122] Patel PJ , Trivedi GR, Shah RK et al. Selenorhizobacteria: as biofortification tool in sustainable agriculture. *Biocatal Agric Biotechnol.* 2018;14:198–203.

[123] Zhalnina K , Louie KB, Hao Z et al. Dynamic root exudate chemistry and microbial substrate preferences drive patterns in rhizosphere microbial community assembly. *Nat Microbiol.* 2018;3:470–80. 29556109 10.1038/s41564-018-0129-3

[124] Sura-de Jong M , Reynolds RJB, Richterova K et al. Selenium hyperaccumulators harbor a diverse endophytic bacterial community characterized by high selenium resistance and plant growth promoting properties. *Front Plant Sci.* 2015;6:113. 25784919 10.3389/fpls.2015.00113 PMC4345804

[125] Lindblom SD , Wangeline AL, Barillas JRV et al. Fungal endophyte *Alternaria tenuissima* can affect growth and selenium accumulation in its hyperaccumulator host *Astragalus bisulcatus*. *Front Plant Sci.* 2018;9:1213. 30177943 10.3389/fpls.2018.01213 PMC6109757

[126] Trivedi G , Patel P, Saraf M. Synergistic effect of endophytic selenobacteria on biofortification and growth of *Glycine max* under drought stress. *S Afr J Bot.* 2020;134:27–35.

[127] Xu X , Cheng W, Liu X et al. Selenate reduction and selenium enrichment of tea by the endophytic *Herbaspirillum* sp. strain WT00C. *Curr Microbiol.* 2020;77:588–601. 30963199 10.1007/s00284-019-01682-z PMC7075828

[128] Wang T , Yang S, Chen Y et al. Microbiological properties of two endophytic bacteria isolated from tea (*Camellia sinensis L*.). *Acta Microbiol Sin.* 2014;54:424–32. 25007655

[129] Guo QX , Liu L, Liu JT et al. Plant sex affects plant-microbiome assemblies of dioecious *Populus cathayana* trees under different soil nitrogen conditions. *Microbiome.* 2022;10:191. 36333709 10.1186/s40168-022-01387-9 PMC9636617

[130] Stringlis IA , Yu K, Feussner K et al. MYB72-dependent coumarin exudation shapes root microbiome assembly to promote plant health. *Proc Natl Acad Sci U S A.* 2018;115:5213–22. 10.1073/pnas.1722335115 PMC5984513 29686086

[131] Chen QW , Yu L, Chao W et al. Comparative physiological and transcriptome analysis reveals the potential mechanism of selenium accumulation and tolerance to selenate toxicity of *Broussonetia papyrifera*. *Tree Physiol.* 2022;42:2578–95. 35899437 10.1093/treephys/tpac095

[132] Cheng BX , Wang CX, Chen FR et al. Multiomics understanding of improved quality in cherry radish (*Raphanus sativus* L. *var. radculus pers*) after foliar application of selenium nanomaterials. *Sci Total Environ.* 2022;824:153712. 35149065 10.1016/j.scitotenv.2022.153712

